# Delayed severe hemothorax caused by a staple line of a bullectomy performed 11 years earlier

**DOI:** 10.1186/s40792-023-01775-9

**Published:** 2023-11-07

**Authors:** Takashi Yamashita, Katsuyuki Asai

**Affiliations:** 1 General Thoracic and Breast Surgery, Iwata City Hospital, 512-3, Ohkubo, Iwata, Shizuoka 438-8550 Japan; 2 General Thoracic Surgery, Hamamatsu Medical Center, 328, Tomitsuka, Hamamatsu, Shizuoka 432-8580 Japan

**Keywords:** Hemothorax, Staple, Ramucirumab, Cilostazol, Bullectomy, Complication, Thoracotomy

## Abstract

**Background:**

At present, relatively few lung surgeries are performed without endostaplers. Although there are few staple-related adverse events, severe events must be shared to improve safety.

**Case presentation:**

A 74-year-old male suddenly collapsed and was transferred to the Emergency Rescue department. He had shock vitals and contrast-enhanced CT revealed extensive right hemothorax with contrast leakage. He lost consciousness and tension massive hemothorax was suspected. We performed emergency thoracotomy at two sites and were able to achieve hemostasis and save the patient. Upon examining the patient's medical history after his condition stabilized, it was revealed that he was a lung cancer patient who was taking ramucirumab and cilostazol. In addition, the CT scan taken one month before onset revealed the bleeding site of the fifth intercostal artery were almost contact with the staple line from a prior right spontaneous pneumothorax surgery that was performed 11 years previously, which was seemed to damage the intercostal artery.

**Conclusion:**

Despite the difficulty in achieving hemostasis due to drug administration history, we successfully treated a case of remote period massive hemothorax attributed to staples, thereby saving the patient. When using drugs that increase the risk of bleeding events, it may be important to consider the position of the staple line while assessing the risk. In the emergent or ICU setting, if the initial incision is not effective, the placement of a new second incision may be valuable.

## Background

At present, relatively few lung surgeries are performed without endostaplers. According to a report on staple-related adverse events, postoperative staple-related bleeding had a frequency of 0.04% [1]. Although the occurrence of bleeding is very low, it can lead to severe consequences. Recent reports [2, 3] have highlighted the need for caution, particularly in cases where wedge resection or partial resection is performed, which results in minimal excised lung tissue and less residual pleural space. Such cases necessitate attention due to potential contact between the staple line and surrounding tissues. This concern is even more pronounced when performing bullectomy during pneumothorax surgery, which has led to cases of postoperative bleeding due to chest wall damage when using absorbable tissue reinforcement materials, resulting in recalls [4]. In both scenarios, taking measures to reduce postoperative complications by devising strategies when performing surgery in areas with small lung resection volumes or high respiratory mobility is crucial. This approach can potentially prevent postoperative acute-phase bleeding complications.

Herein, we report a case in which massive hemothorax attributed to the staple line occurred 11 years after pneumothorax surgery.

## Case report

**Fig. 1:**
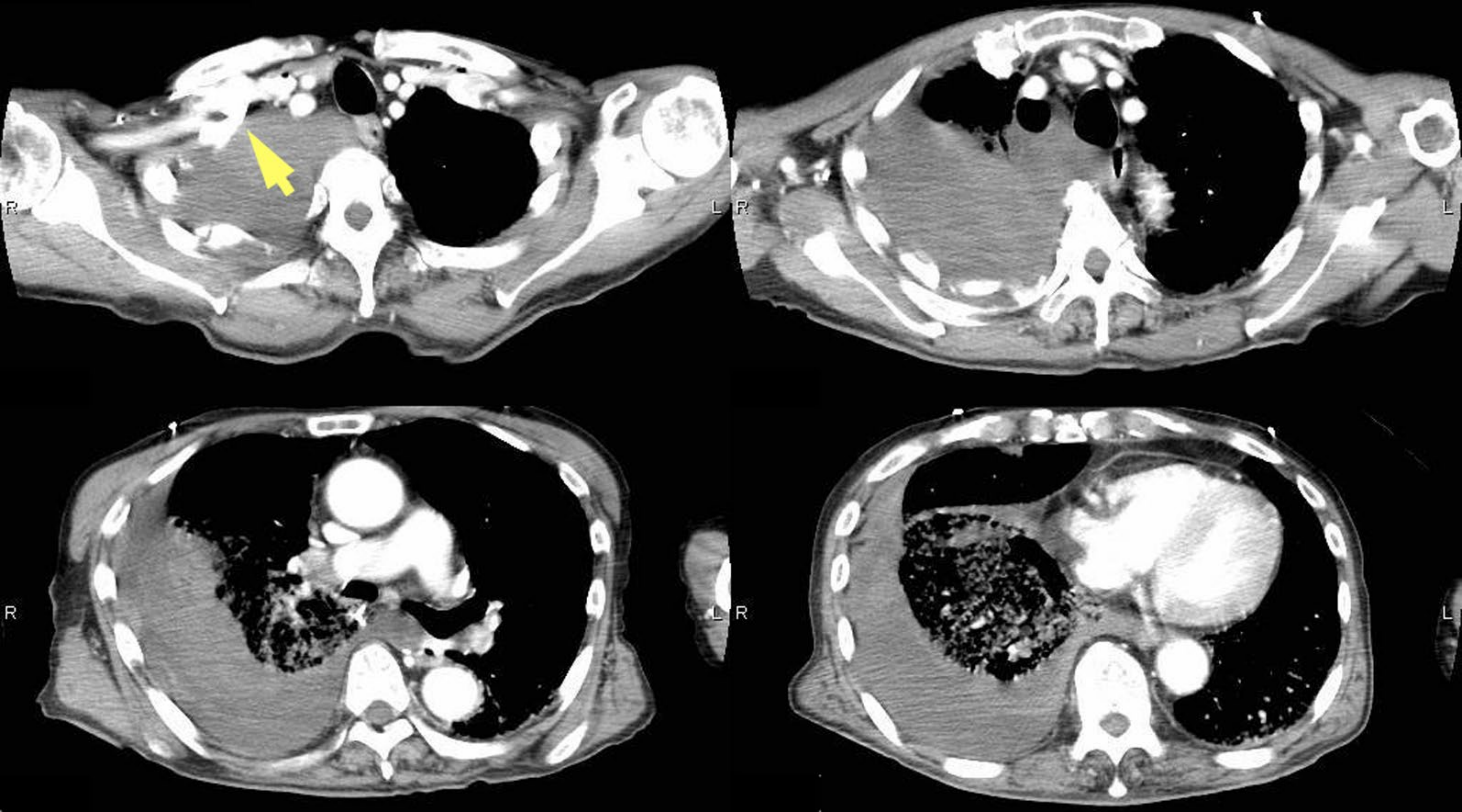
Contrast-enhanced CT. Contrast-enhanced CT reveals massive pleural effusion with high CT attenuation, suggestive of a blood component, along with contrast agent leakage within the pleural cavity. Initially, the source of bleeding was suspected to be the right subclavian artery (yellow arrow)

A 74-year-old male, 167 cm in height and 55 kg in weight (BMI 19.7), suddenly collapsed and was transferred to the Emergency Rescue department. On admission, his blood pressure was 48/31 mmHg, heart rate was 120 beats/min, SpO2 99% (5L mask), and he presented as E4V4M6. Despite extracellular fluid resuscitation, there was no response, prompting immediate initiation of the massive transfusion protocol with pumping blood transfusion. Contrast-enhanced CT revealed extensive right hemothorax with contrast leakage (Fig. 1).

Just before the placement of the drain, he lost consciousness, and the radial artery pulse became impalpable. The patient had concomitant distension of the jugular vein; thus, tension massive hemothorax was suspected, prompting urgent decompression drainage. Initially, we suspected bleeding from the subclavian artery and placed a drain in the second intercostal space on the midclavicular line, considering the possibility of conversion to open thoracotomy in the supine position.

Decompression was achieved, however, consciousness did not return. We expanded the incision anteriorly in the second intercostal space and performed emergency open thoracotomy; however, there was no bleeding from the thoracic apex, and the subclavian artery was intact. On the dorsal side, a large hematoma had accumulated, obscuring the bleeding point. An additional lateral thoracotomy in the fifth intercostal space was performed urgently while maintaining the supine position. Although the hematoma was significantly drained, the right lower lobe remained tense and was immobile, resulting in poor visibility. Upon re-observing the intracavity from the second intercostal incision, we observed sustained pulsatile bleeding from the fifth intercostal artery near the vertebral body. We cauterized this bleeding, which restored blood pressure and obviated the need for pumping. When we reviewed the CT for a moment before thoracotomy, it was initially thought that the diffused contrast agent was flowing out from the right subclavian artery. However, it turned out that this was the distal end of the contrast, and the actual upstream source was the contralateral side near the vertebral body, in other words, the intercostal artery.

**Fig. 2:**
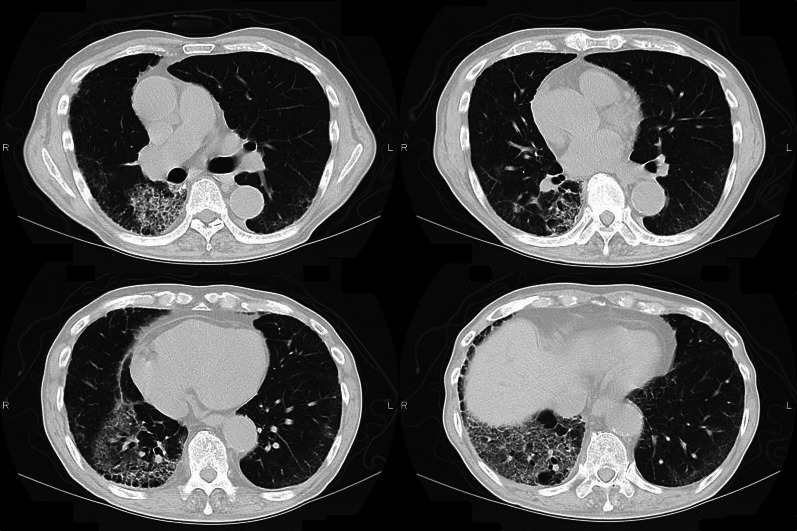
CT; one month before the onset of hemothorax. The invasive mucinous adenocarcinoma occupies a significant portion of the right lower lobe. The mobility of the lung is extremely limited

After his condition stabilized, we learned that the patient was undergoing second-line chemotherapy with docetaxel (DTX) + ramucirumab for invasive mucinous adenocarcinoma of the right lower lobe (Fig. 2). The last dose had just been administered for the third cycle 17 days before the hemothorax onset. He also had a history of stroke and was taking cilostazol started 8 years ago.

As blood pressure increased, bleeding from the incision edge escalated. Given the patient's medical history, it was judged that natural hemostasis would be difficult to achieve. Consequently, we performed surgery under general anesthesia, ligating the dissected lateral thoracic artery, followed by intracavity cleansing and closure. The patient's overall condition stabilized. From visit to the end of surgery, a total of 5.5 h had elapsed, during which fluid and blood transfusions consisting of 12 units of red blood cell (RBC) A type, 10 units of RBC mismatched O type, 16 units of fresh frozen plasma, 20 units of platelets, and 3850 mL of extracellular fluid had been administered.

**Fig. 3:**
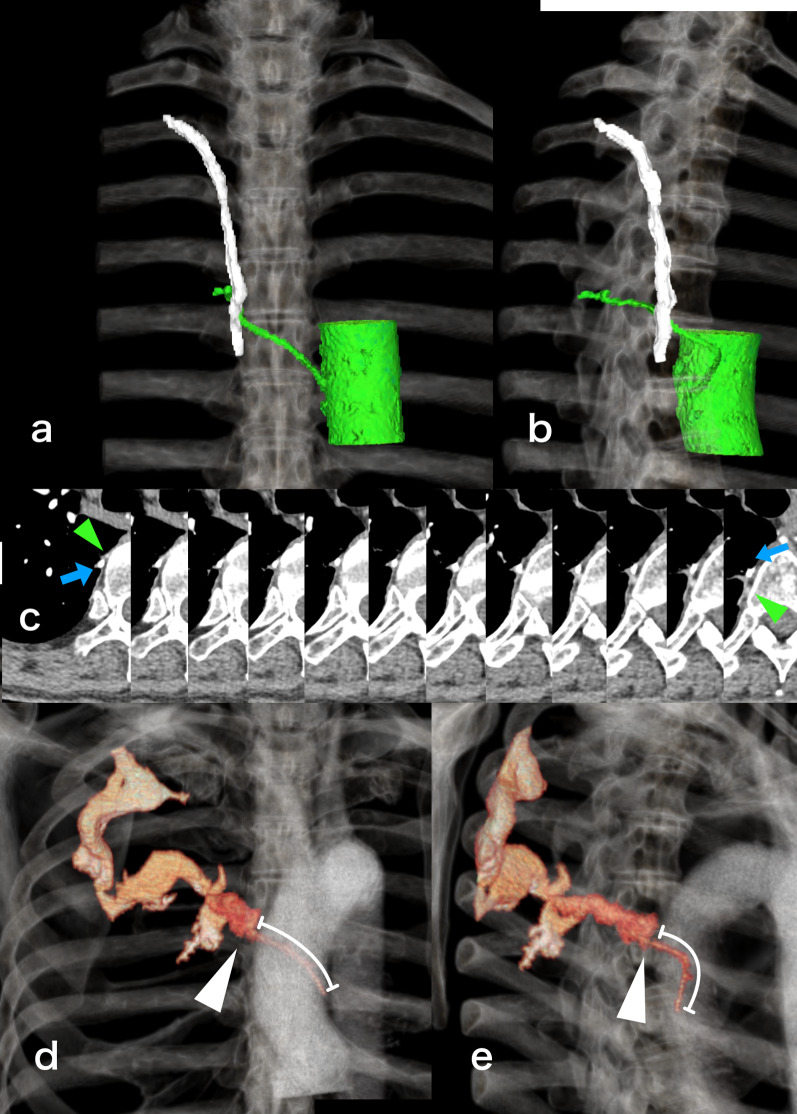
Relationship between the bleeding site of the fifth intercostal artery and the staple line. **a** Frontal view, **b** right anterior oblique view showing the relationship between the staple line (white) and the fifth intercostal artery (light green) reconstructed based on the CT scan from one month before hemothorax onset. **c** Sequential axial images showing the staple line (blue arrow) and the fifth intercostal artery originating from the aorta (light green arrowhead) crossing each other. **d** Frontal view, **e** right anterior oblique view of reconstructed contrast-enhanced CT scan at onset showing the disrupted fifth intercostal artery and the contrast medium leaking into the thoracic cavity. The intact part of the fifth intercostal artery indicated by white line, and the bleeding point indicated by white arrowhead. The staple line from the past pneumothorax surgery aligns with the fifth intercostal artery, and the bleeding point was exactly the same location. Despite a shock vital with BP 50/30 and HR 120, an approximate blood loss of 40 mL/min was measured. All of the contrast medium leaked out at the bleeding point, and the peripheral fifth intercostal artery beyond the bleeding point was not visualized

The patient's medical history was reviewed in detail postoperatively. The CT scan taken one month before onset revealed that the bleeding site of the fifth intercostal artery were almost contact with the staple line from a prior right spontaneous pneumothorax surgery that was performed 11 years before (Fig. 3a–c). Comparing with the contrast-enhanced CT at onset, the bleeding point was consistent with the crossing point of the staple line (Fig. 3d, e). The surgical record at the time revealed that right S6 bullectomy was performed using an endostapler once (Echelon Flex Endopath with a 60 mm blue cartridge; Ethicon, Johnson & Johnson, NJ, USA) without reinforcements or covering materials.

**Fig. 4:**
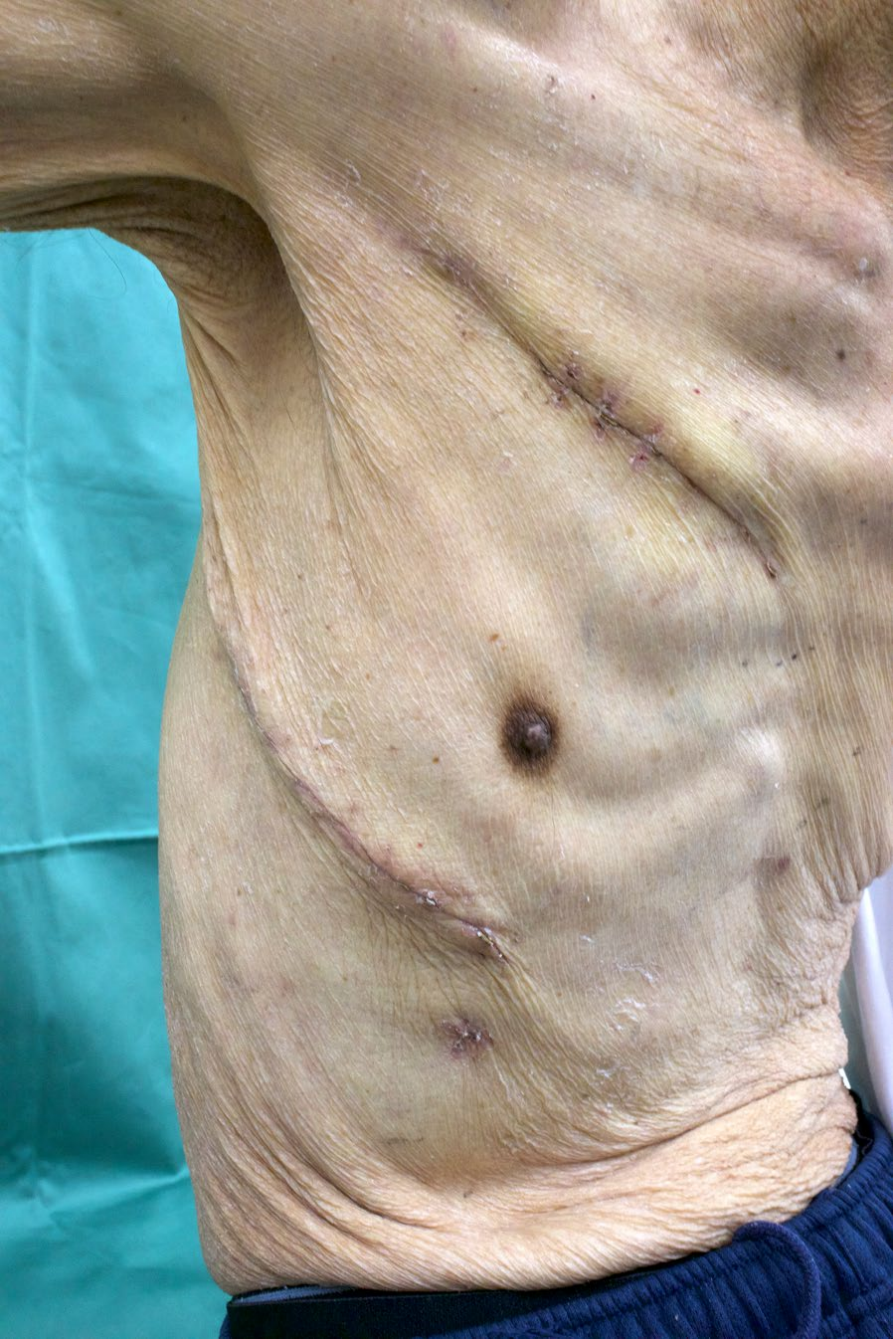
Postoperative scars. Anterior thoracotomy in the second intercostal space and lateral thoracotomy in the fifth intercostal space are used

Postoperative progress was favorable, and the patient was discharged on postoperative day 13 (Fig. 4). Chemotherapy was resumed on postoperative day 26, using DTX monotherapy to avoid bleeding events.

## Discussion

This case represents a life-saving example of right-sided massive hemothorax associated with a staple line. A comparison with past CT scans revealed the involvement of staples in the bleeding. However, despite staples being used extensively worldwide in thoracic surgery, there are few reports of staples causing bleeding in the remote postoperative period. Direct injury to the chest wall or blood vessels solely due to staples is unlikely, especially beyond the postoperative acute phase. Nevertheless, studies on granulation tissue at the staple site have reported varying degrees of granulation [5], which may not be discernible by CT imaging. Considering the patient's cancerous state and ongoing chemotherapy, the decrease in intervening granulation tissue and increasing tissue fragility may have played a role in the progression of the disease. Additionally, this case had not used any staple line reinforcement in the past procedure. Anticipating contact with surrounding tissue, using reinforcements or absorbable materials might increase granulation tissue and prevent hemorrhagic events in the remote postoperative period. However, reinforcing materials must be used in consideration of the risk of bleeding during the acute phase.

The patient had been administered ramucirumab and cilostazol that promote bleeding. Using the SYNAPSE VINCENT 3D volume analyzer (Fuji Medical Systems, Tokyo, Japan), a volume increase of approximately 20 mL of contrast agent leakage was measured during the 30 s between the arterial and equilibrium phases of contrast-enhanced CT (Fig. 3d, e). At that time, the patient's vital signs were approximately BP 50/30 mmHg and HR 120/min, indicative of shock. Under this shock vital, hemostasis could have been achieved spontaneously; thus, the effects of cilostazol and ramucirumab were considered to have contributed to the patient’s condition. However, neither drug on its own is expected to result in massive hemothorax; thus, we suspected a combined effect with the staple line.

To investigate the involvement of staples alone in the development of postoperative hemothorax, we conducted an English-language case report-specific search on PubMed regarding hemothorax caused by staples. Several case reports were found (Table 1), but there are no reports of cases that develop hemothorax caused by staples with any drugs increasing risk of hemorrhage, and as late as the present case. Considering the widespread use of staples, although cases like this one are extremely rare, it can be said that adverse events caused by staples may occur also in the remote period especially in the presence of hemorrhagic status. When using drugs that increase bleeding propensity, it may be important to recognize the potential elevation of bleeding risk from intrathoracic bleeding due to staples. Additionally, 3 cases including our case in Table 1 had bleeding originating from the paravertebral intercostal artery. This suggests that extra attention may be required regarding the dorsal staple lines.

**Table 1:** Previous cases of hemothorax associated with staple lines

No.	Author/year	Age	Sex	Procedure	Device	Time to onset	Bleeding lesion
1	Motoyama [6]/2009	74	F	Lobectomy	n/a	11 days	Right intercostal artery near the vertebra
2	Kanai [7]/2012	58	F	Wedge resection	Duet TRS	1 day	Right intercostal artery of the lateral chest wall
3	Hayashi [8]/2018	69	M	Bullectomy during lobectomy	Endo GIA Reinforced Reload with Tri-Staple Technology	1 h	Left lung surface near the staple line
4	Negishi [9]/2019	60	M	Segmentectomy	Echelon	40 days	Right intercostal artery near the vertebra
5	Yamano [3]/2020	80	M	Wedge resection	Powered Echelon	4 h	Right parietal pleura
6	Yamaji [10]/2022	61	M	Wedge resection	n/a	6 h	Aorta (left)
7	Our case	74	M	Bullectomy	Echelon Flex Endopath	11 years	Right intercostal artery near the vertebra

Although the emergency thoracotomy was unplanned, it resulted in two separate non-contiguous thoracotomies, ultimately contributing to the patient's survival. An anterior thoracotomy alone would not have allowed removal of the hematoma or identification of the bleeding point, and a lateral thoracotomy alone would have obstructed lung movement, preventing identification of the bleeding point. The need to explore the subclavian artery area near the apex of the pleural cavity also influenced the situation. In cases where the bleeding point is not definite, it is important to consider placing a large fourth intercostal incision, akin to the standard procedure for left emergency thoracotomy, that allows for easier expansion of the incision and transition to clamshell thoracotomy. However, since performing a large thoracotomy requires sufficient personnel, attempting a small incision in cases with limited initial personnel is conceivable. In that case, if the initial incision is not effective, the placement of a second incision is valuable, which can offer an alternate perspective. Based on our experience, we would like to emphasize that a supine lateral thoracotomy is not suitable for exploring the pleural cavity.

## Conclusion

Despite the difficulty in achieving hemostasis due to drug administration history, we successfully treated a case of remote period massive hemothorax attributed to staples. When using drugs that increase the risk of bleeding events, it may be important to consider the position of the staple line while assessing the risk. In the emergent or ICU setting, if the initial incision is not effective, the placement of a new second incision may be valuable.

## Data Availability

Not applicable.

## Abbreviations

BMI: Body mass index
CT: Computed tomography
DTX: Docetaxel
ICU: Intensive care unit
RBC: Red blood cell
